# High-Resolution Structural and Functional Assessments of Cerebral Microvasculature Using 3D Gas ΔR_2_*-mMRA

**DOI:** 10.1371/journal.pone.0078186

**Published:** 2013-11-04

**Authors:** Chien-Hsiang Huang, Chiao-Chi V. Chen, Tiing-Yee Siow, Sheng-Hsiou S. Hsu, Yi-Hua Hsu, Fu-Shan Jaw, Chen Chang

**Affiliations:** 1 Institute of Biomedical Engineering, National Taiwan University, Taipei, Taiwan; 2 Institute of Biomedical Sciences, Academic Sinica, Taipei, Taiwan; University of Louisville, United States of America

## Abstract

The ability to evaluate the cerebral microvascular structure and function is crucial for investigating pathological processes in brain disorders. Previous angiographic methods based on blood oxygen level-dependent (BOLD) contrast offer appropriate visualization of the cerebral vasculature, but these methods remain to be optimized in order to extract more comprehensive information. This study aimed to integrate the advantages of BOLD MRI in both structural and functional vascular assessments. The BOLD contrast was manipulated by a carbogen challenge, and signal changes in gradient-echo images were computed to generate ΔR_2_* maps. Simultaneously, a functional index representing the regional cerebral blood volume was derived by normalizing the ΔR_2_* values of a given region to those of vein-filled voxels of the sinus. This method is named 3D gas ΔR_2_*-mMRA (microscopic MRA). The advantages of using 3D gas ΔR_2_*-mMRA to observe the microvasculature include the ability to distinguish air–tissue interfaces, a high vessel-to-tissue contrast, and not being affected by damage to the blood–brain barrier. A stroke model was used to demonstrate the ability of 3D gas ΔR_2_*-mMRA to provide information about poststroke revascularization at 3 days after reperfusion. However, this technique has some limitations that cannot be overcome and hence should be considered when it is applied, such as magnifying vessel sizes and predominantly revealing venous vessels.

## Introduction

Abnormal structure and function of cerebral microvessels, including arterioles, venules, and capillaries, are pathological features that have been increasingly recognized in brain disorders [1]. Identifying unusual microvascular changes may be useful in the differential diagnosis and prognosis of various diseases, including cancer [2] and ischemic [3], [4] and neurodegenerative [5]–[7] diseases. Methods for evaluating the microvascular structure and function are therefore necessary to facilitate accurate diagnosis, provide insights for therapy development, and for monitoring therapeutic responses in brain disorders.

Our group previously proposed a method for simultaneously visualizing the microvascular architecture and obtaining a functional vascular index, called 3D ΔR_2_-based microscopic magnetic resonance (MR) angiography (3D ΔR_2_-mMRA) [8]. Although 3D ΔR_2_-mMRA has been successfully used to detect arterioles and venules and obtain the microvascular cerebral blood volume (CBV), the application of contrast agents in MR angiography was found to have substantial limitations in a diseased status, such as in the presence of damage to the blood–brain barrier (BBB), which is a common pathological sign in many brain diseases. Such damage tends to result in contrast agent extravasating via the leaky BBB, which causes inaccurate visualization and estimation of the vasculature [9]–[11]. Furthermore, the use of iron-based contrast agents is problematic due to their availability, cost, and safety [12].

As alternatives to using contrast agents, recent studies have exploited the intrinsic blood oxygen level-dependent (BOLD) contrast for observing microvessels [13]. Some studies have demonstrated the detectability of BOLD contrast in the microvasculature [14], [15]. Furthermore, the BOLD response to a gas challenge (altered fractions of O_2_ and CO_2_ in the inspired gas) has been shown to have the potential to reveal the detailed vasculature [16],[17]. Several angiographic techniques have been proposed using the BOLD contrast with or without a gas challenge [16], [18]–[21], and some studies have enhanced the BOLD contrast using susceptibility-weighted imaging, which involves applying additional phase-contrast filtering in gradient-echo imaging [16], [20], [21].

While these previous angiographic methods offer appropriate visualization of the cerebral vasculature, the methods remain to be optimized in order to extract more comprehensive information. Importantly, the BOLD contrast is considered a quantifiable measure of vascular functions when normalized and calculated appropriately [22]. For example, a functional index representing the regional cerebral blood volume (rCBV) was derived by normalizing the ΔR_2_* values of a given region to those of vein-filled voxels of the sinus [23], [24] in response to a gas challenge. Combining simultaneous quantitative functional assessments with visualization of the cerebral vasculature is likely to yield a highly advantageous angiographic method.

The present study aimed to integrate the advantages of BOLD MR imaging (MRI) in both structural and functional vascular assessments. 3D high-resolution gradient-echo imaging (with a spatial resolution 50×50×73 microns) was applied for detecting the BOLD response to a carbogen challenge in order to generate ΔR_2_* maps. Visualization of the cerebral microvasculature and quantification of rCBV were simultaneously achieved after volume rendering of the ΔR_2_* maps. For convenience, the method used in this study is herein named 3D gas ΔR_2_*-mMRA. To demonstrate the utility of the 3D gas ΔR_2_*-mMRA method, we compared it with both MR venography and 3D ΔR_2_-mMRA. Its potential usefulness was further demonstrated by applying it to a stroke rat model to study poststroke revascularization at 3 days after reperfusion.

### Theory

The signal intensity of T_2_*-weighted images (T_2_*WIs) is determined as
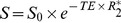
(1)where S_0_ is the zero-echo-time (TE) signal and R_2_* is the transverse relaxation rate. Since deoxyhemoglobin influences the field homogeneity as a paramagnetic contrast agent, the deoxyhemoglobin concentration has a linear effect on R_2_* [19]:
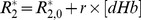
(2)where R2,0* is the relaxation rate of blood without deoxyhemoglobin, r is the relaxivity constant of deoxyhemoglobin, and [dHb] is the deoxyhemoglobin concentration described by
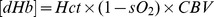
(3)where Hct is the hematocrit. The ΔR2* value induced by gas challenges is related to the changes in the deoxyhemoglobin concentration [25] according to
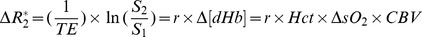
(4)where S1 and S2 are the signal intensities of T2*WIs while inhaling two gases given sequentially. The rCBV can be derived as [23]

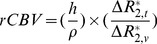
(5)where ρ is the density of brain tissue ( = 1.04 g/mL), h = (1–Hct)/(1–r×Hct) is a term to correct for the hematocrit being higher in larger vessels than in small vessels (r = 0.85 according to Phelps et al. [26]), and ΔR2,t* and ΔR2,v* are the transverse relaxation rate changes of tissue in the region of interest (ROI) and vein-filled voxels (i.e., sinus), respectively.

## Materials and Methods

### Subjects

Twelve male Sprague-Dawley rats purchased from the National Laboratory Animal Center of Taiwan were used in this study. Each plastic cage housed three animals with free access to food and water, and the experiments were performed when they were 8 weeks old. The housing environment had a 12∶12-h light:dark cycle with controlled humidity and temperature. The rats were kept in a specific-pathogen-free environment throughout the study. All experimental procedures were approved by the Institute of Animal Care and Utilization Committee at Academia Sinica, Taipei, Taiwan. MRI experiments were performed on six naive rats and one rat with ischemic stroke. Additional five rats were used for measuring physiological parameters.

### Stroke Model for Studying Poststroke Revascularization

3D gas ΔR_2_*-mMRA was applied in a stroke rat model of middle cerebral artery occlusion (MCAO) to study poststroke revascularization. The three-vessel occlusion model was induced using a previously described procedure [27]. In brief, the right middle cerebral artery was transiently ligated, and then the common carotid arteries on both sides were also occluded using nontraumatic aneurysm clips. After 60 min, the reperfusion was accomplished by releasing all of the arterial occlusions. The rectal temperature of anesthetized rats was maintained at 37.0±0.5°C (mean±SD) using a homeothermic blanket (Harvard, Holliston, MA, USA). Three days after surgery the animals with MCAO underwent MRI experiments because the poststroke revascularization is most evident at this time point after reperfusion (please refer to Figure S1).

### Blood Gas, Blood Pressure, and Oxygen Saturation Measurements

Due to experimental difficulties, the physiological parameters were monitored in a separate batch of age-matched control rats (*n* = 5) that were prepared identically to those used in the imaging studies. The heart rate, blood pressure, sO_2_, and partial pressures of arterial oxygen (pO_2_) and CO_2_ (pCO_2_) were monitored under the inhalation of air, oxygen (100% O_2_), or carbogen (5% CO_2_ +95% O_2_). A sensor was mounted along the axis of the rat right foot, and a photodiode was placed on the shaved ventral side to record the heart rate (MouseOx, STARR Life Sciences, Oakmont, PA, USA). One femoral artery was cannulated with PE-50 tubing for monitoring the blood pressure, while blood samples were drawn from the other for blood gas analysis of pO_2_, pCO_2_, and sO_2_ using a blood gas analyzer (ABL5, Radiometer America, Westlake, OH, USA). The drawn volume was 0.1 mL each time. The physiological data are summarized in Table 1.

**Table 1:** Physiological parameters in various inhalation conditions.

	Heart rate (/min)	Blood pressure (mmHg)	pO_2_ (mmHg)	pCO_2_ (mmHg)	sO_2_ (%)
Air	353.2±18.9	90.2±3.6	89.8±9.2	41.8±5.8	97.4±0.6
100% O_2_	340.2±21.9*	94.8±5.6	328.8±29.3**	45.4±11.3	100±0**
5% CO_2_+95% O_2_	366±8.5	93.6±12.3	359.6±30.2**	56±6.2**	100±0**

* P<0.05 relative to air.

** P<0.01 relative to air.

### MRI Experiments

Rat MRI experiments were performed on a 7-T PharmaScan 70/16 MR scanner (Bruker, Germany) with an active shielding gradient (30 G/cm in 80 µs). Images were acquired using a 72-mm birdcage transmitter coil and a separate quadratic surface coil for signal detection. Each rat was initially anesthetized with 5% isoflurane flowing in air at 2 L/min. Once the animal was fully anesthetized, the isoflurane was maintained at 1.5∼2.0% to minimize anesthesia-induced hemodynamic fluctuations. The rat was allowed to breathe spontaneously throughout the experiment. The rectal temperature was monitored and maintained at 37.0±0.5°C by a water circulation system. To determine ΔR_2_* for microvasculatural characterization, T_2_*WIs were acquired under the inhalation of air, oxygen, or carbogen delivered through a nose cone. An interval of 15 min was imposed between gas changes to allow complete gas exchange. Each T_2_*WI was acquired using a 3D gradient-echo sequence with flow compensation and the following parameters: matrix size = 256×256×96 with zero-filling to 512×512×192, field of view (FOV) = 2.56×2.56×1.4 cm^3^, repetition time (TR) = 90 ms, TE = 25 ms, flip angle = 15°, bandwidth = 9 kHz, averages = 2, and total acquisition time = 73 min. The results of 3D gas ΔR_2_*-mMRA were compared with those of MR venography, which corresponded to the T_2_*WIs acquired during air inhalation. 3D gas ΔR_2_*-mMRA was also compared with 3D ΔR_2_-mMRA under identical resolution and geometrical settings. 3D ΔR_2_-mMRA employed a 3D fast spin-echo sequence before and after the intravenous injection of monocrystalline iron-oxide nanoparticles (MION) at a dose of 20 mg Fe/kg. The acquisition parameters were as follows: TR = 1500 ms, TE_eff_ = 82 ms, bandwidth = 50 kHz, echo-train length = 32, averages = 4, matrix size = 256×256×96 with zero-filling to 512×512×192, FOV = 2.56×2.56×1.4 cm^3^, and acquisition time = 76 min.

### Use of ΔR_2_* Maps to Reconstruct 3D gas ΔR_2_*-mMRA

3D gas ΔR_2_*-mMRA was reconstructed according to the procedure used for 3D ΔR_2_-mMRA [8]. In brief, the two sets of T_2_*WIs acquired under the inhalation of two gas types were coregistered in MRVision (MRVision Company, Winchester, MA, USA) on a pixel-by-pixel basis to produce a ΔR_2_* map according to formula (4). The boundary of the soft tissue was manually selected on T_2_*WIs acquired for carbogen inhalation on a slice-by-slice basis, and then applied to the ΔR_2_* map to exclude nonbrain tissue. The segmented high-resolution ΔR_2_* maps were reconstructed into a 3D gas ΔR_2_*-mMRA with the volume rendering utility of a commercial 3D visualization platform (Avizo software, TGS, San Diego, CA, USA).

### MRI Data Analysis

A 1-mm-thick slab composed by 20 axial slices was selected for rCBV analysis. The center of the slab was aligned to the anterior commissure. ROIs in the sinus, cortex, striatum, and hippocampus were defined manually according to a brain atlas. The ΔR_2_* value of an ROI was normalized by division by the ΔR_2_* value of the sinus. The rCBV was calculated from the normalized ΔR_2_* based on formula (5). For vessel size and density analysis, the pixels with ΔR_2_* or ΔR_2_ values higher than mean+2×SDs in the selected plane were defined as blood vessels. The observed vessel size was evaluated by the average width of the bright dots in ΔR_2_* or ΔR_2_ maps. Paired t-tests were used to identify any differences in the observed vessel size and density between 3D gas ΔR_2_*-mMRA and 3D ΔR_2_-mMRA.

### Latex Perfusion

After MRI experiments, rats (*n* = 2) were deeply anesthetized with chloral hydrate (450 mg/kg; Sigma, St. Louis, MO, USA) and then perfused transcardially with saline followed by 4% paraformaldehyde. Subsequently, 9.5 mL of red latex or blue latex containing 4 mL of Microfil premixed with 5 mL of diluent and 0.5 mL of curing agent (Flow Tech, Carver, MA, USA) was injected. The red latex was administered through the left carotid artery to label the cerebral arteries, while the blue latex was administered through the jugular veins to label the cerebral veins. After 90 min, when the liquid latex had hardened, the brain was removed, sectioned, and photographed.

## Results

### The Choice of Optimal BOLD Contrast by Gas Challenges

**Figure 1:**
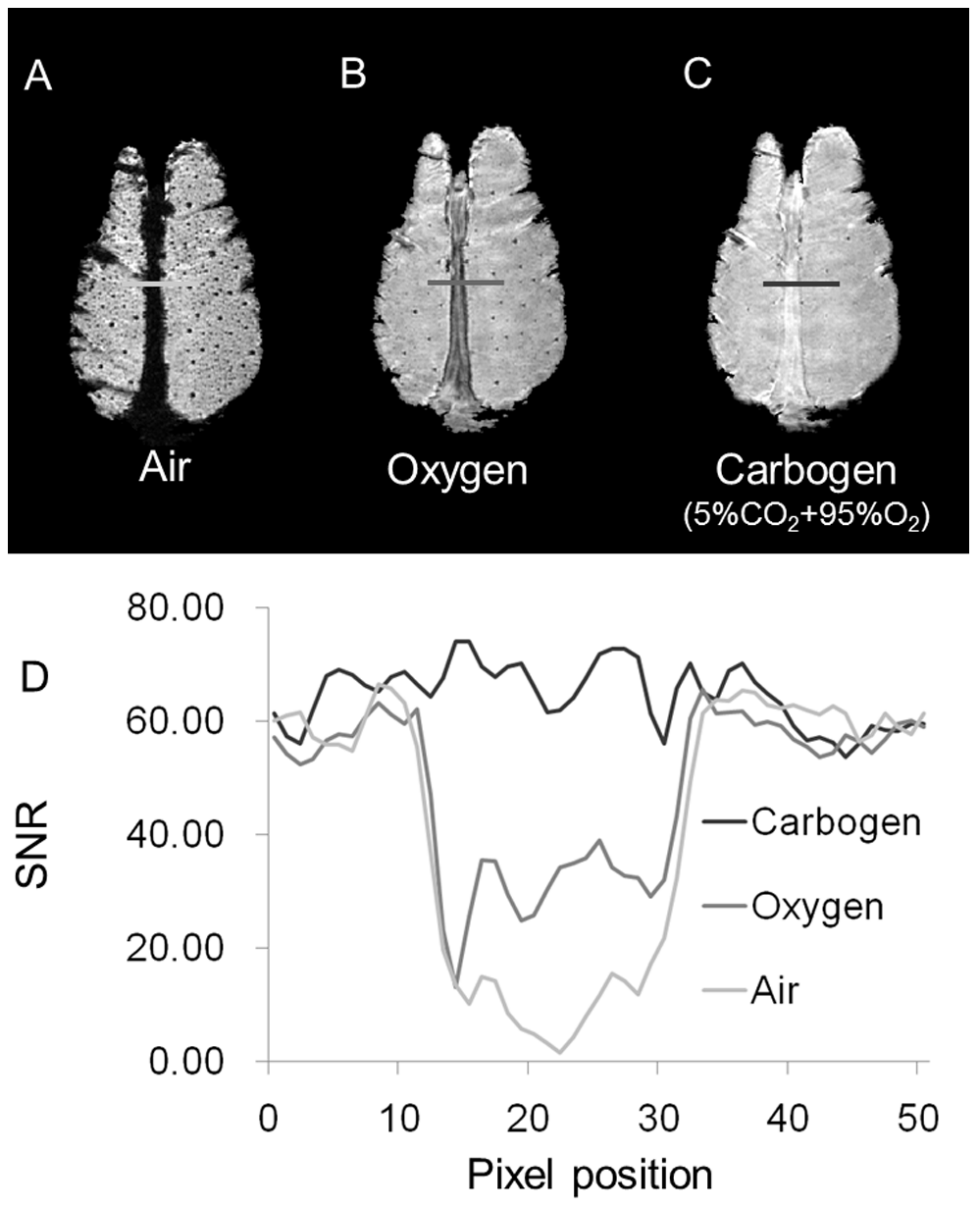
The BOLD contrast for the inhalation of different gases. (A) During air inhalation, the vessels exhibited minimal signal intensities relative to nonvessel brain tissues. (B) During the inhalation of 100% O_2_, fewer hypointensities were evident in the vessels. (C) During the inhalation of carbogen, few hypointensities remained. (D) Comparison of signal profiles of the sinus (horizontal bars in A to C) in the various inhalation conditions. The BOLD contrast was highest for air, followed by oxygen and then carbogen.

The 3D high-resolution T_2_*WIs acquired during the inhalation of air, 100% oxygen, or carbogen exhibited different BOLD contrasts. Slices through the horizontal plane at the brain surface are presented in Figure 1A, 1B, and 1C. During air inhalation (Figure 1A) the vessels had minimal signal intensities relative to nonvessel brain tissues. The cortical penetrating vessels appeared as through-plane hypointense dots on the brain surface, while the sinus appeared as a thick band of hypointensities traversing the brain. During the inhalation of oxygen (Figure 1B) there were fewer hypointensities arising from the vessels owing to the increased saturation of BOLD signals. During carbogen inhalation (Figure 1C) few hypointensities remained in the brain due to greater saturation of the BOLD signals. Figure 1D illustrates the signal profiles of the sinus under the different inhalation conditions; the BOLD contrast was highest for air, followed by oxygen and then carbogen.

### Use of ΔR_2_* Maps to Reconstruct 3D gas ΔR_2_*-mMRA

**Figure 2:**
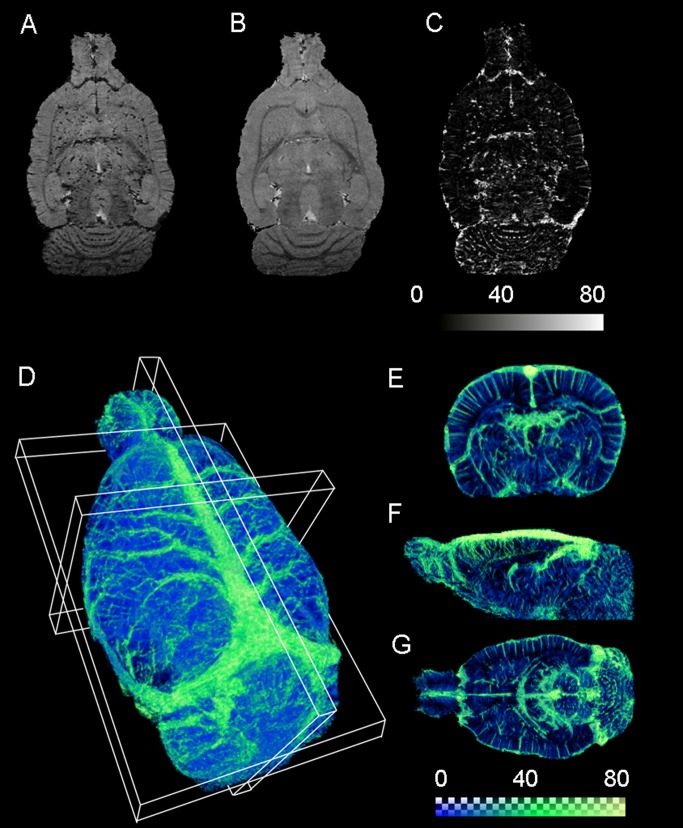
Demonstration of 3D gas ΔR_2_*-mMRA. (A) 3D high-resolution T_2_*WI acquired during the inhalation of air. (B) T_2_*WI acquired during the inhalation of carbogen. (C) ΔR_2_* map computed from the two T_2_*WIs. (D) The reconstructed ΔR_2_* maps, which can be viewed flexibly in various planes. (E) A 1-mm-thick axial view revealing the microvasculature. (F) A sagittal view. (G) A horizontal view. The cortical penetrating vessels are readily distinguishable in each view, and subcortical vessels are also identified in the striatum and hippocampus.

The 3D high-resolution T_2_*WIs acquired during the inhalation of air followed by carbogen are shown in Figure 2A and 2B, respectively. The two T_2_*WIs were used to compute the ΔR_2_* map, as shown in Figure 2C. Figure 2D illustrates that the 3D gas ΔR_2_*-mMRA reconstructed from the ΔR_2_* maps allows flexible viewing in various planes. Brain slices with a thickness of 1 mm that reveal the microvasculature in each of the three orthogonal planes are shown in Figure 2E, 2F, and 2G. The cortical penetrating vessels are readily distinguishable in each view. Subcortical vessels are also evident in the striatum and hippocampus. The rCBV values (*n* = 6) measured in various brain regions–4.34±0.99 mL/100 g in the cortex, 3.61±1.05 mL/100 g in the striatum, and 2.48±1.42 mL/100 g in the hippocampus–are similar to previously reported values obtained by various techniques [28]. The results indicate that 3D gas ΔR_2_*-mMRA offers (1) visualization of the microvasculature at a resolution of 50×50×73 microns and (2) quantification of rCBV.

**Figure 3:**
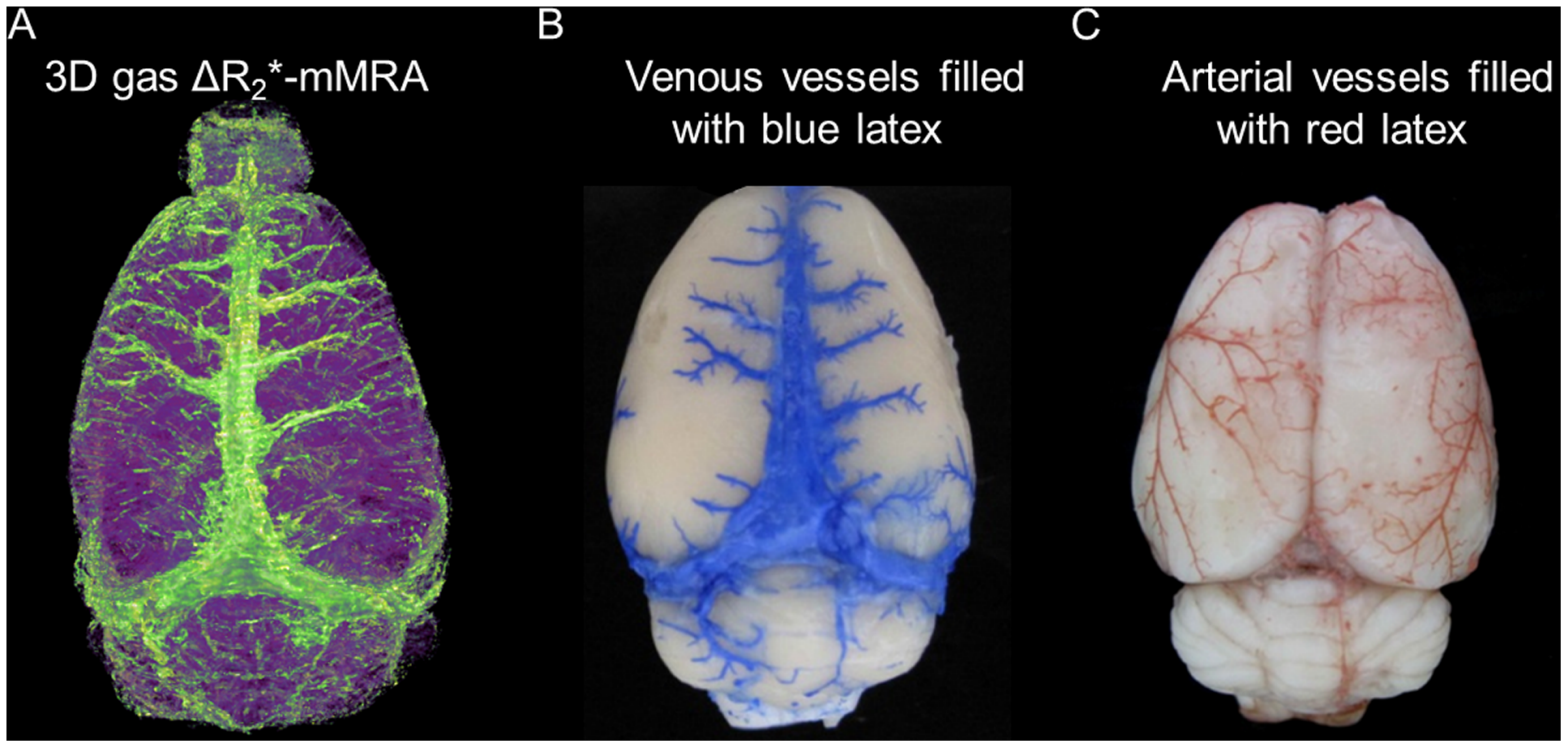
3D gas ΔR_2_*-mMRA visualization compared with venous and arterial vessels labeled by latex. (A) 3D gas ΔR_2_*-mMRA identifies the superior sagittal sinus, superior cerebral veins, and transverse sinus on the brain surface. (B) Venous vessels labeled by blue latex. (C) Arterial vessels labeled by red latex. Comparison of A with B and C indicates that 3D gas ΔR_2_*-mMRA predominately identifies venous vessels.

As shown in Figure 3A, 3D gas ΔR_2_*-mMRA could reveal the superior sagittal sinus, superior cerebral veins, and the transverse sinus on the brain surface. The detected vessels corresponded well to the venous vessels labeled by blue latex in Figure 3B. In contrast, the arterial vessels labeled by red latex shown in Figure 3C were not detected by 3D gas ΔR_2_*-mMRA. This shows that 3D gas ΔR_2_*-mMRA is dominated by the BOLD signals originating from venous vessels.

### Comparison of 3D Gas ΔR_2_*-mMRA and MR Venography

**Figure 4:**
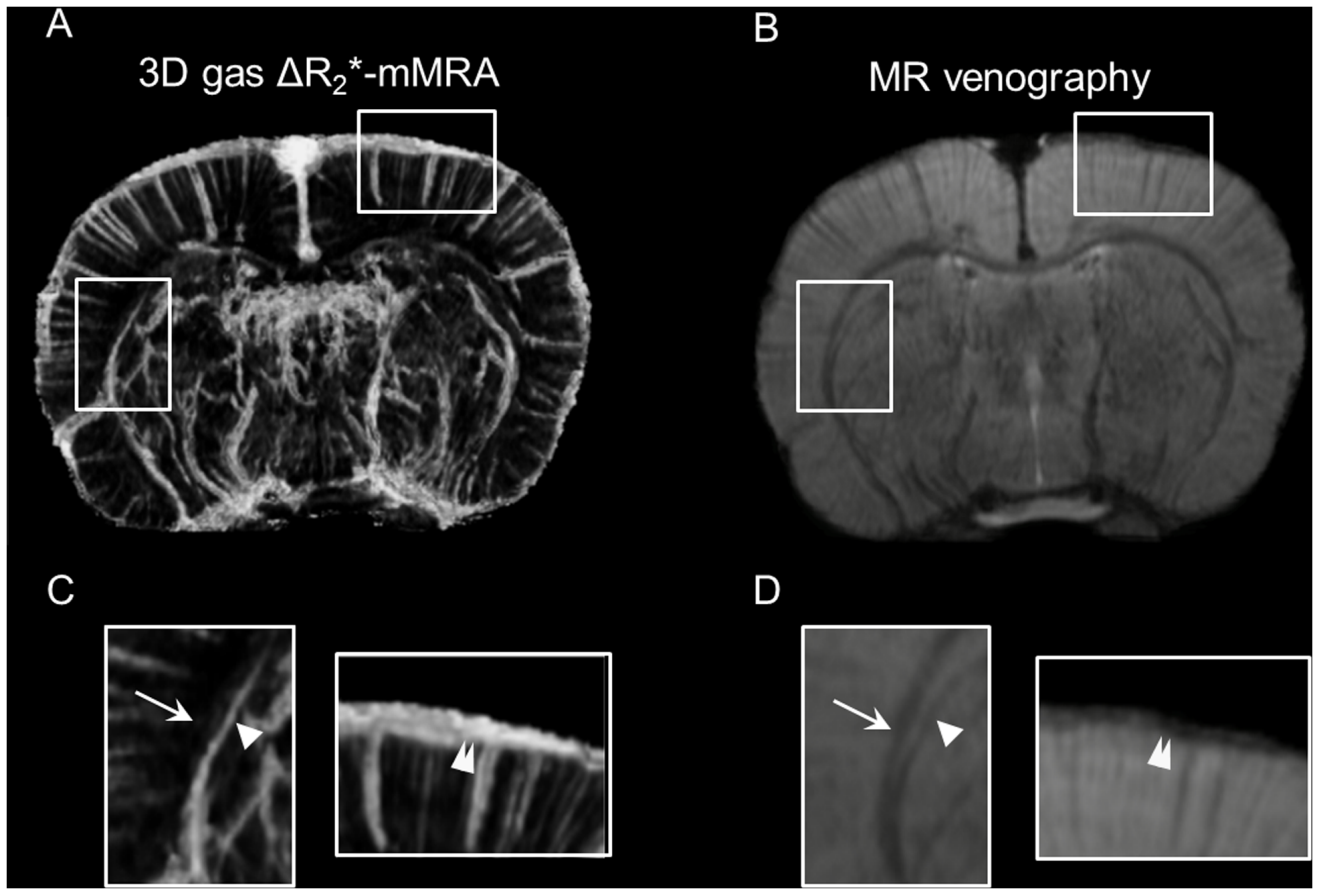
Comparison of 3D gas ΔR_2_*-mMRA and MR venography. (A) An axial slice from 3D gas ΔR_2_*-mMRA. The cortex and white matter are marked by the rectangles and magnified in C. (B) MR venography with identical geometrical settings and ROIs. (C) 3D gas ΔR_2_*-mMRA allows the vessels to be readily distinguished at air–tissue interfaces and along the white matter. The arrow, arrowhead, and double-arrowhead indicate the locations of the external capsule, a vein near external capsule, and a vein at air–tissue interfaces, respectively. (D) In MR venography, the signal dephasing near air–tissue interfaces and white matter obscures the blood vessels.

Axial slices obtained by 3D gas ΔR_2_*-mMRA and MR venography with identical resolution and geometrical settings in the same animal are shown in Figure 4A and 4B, respectively. The microvessels of the cortical and subcortical areas were identified by both angiographic techniques, since the same BOLD effect was employed as the signal source. However, the vessels revealed by MR venography lack quantitative information about cerebral vascular characteristics, while those identified by 3D gas ΔR_2_*-mMRA carry information on the regional rCBV. Additionally, signal dephasing near air–tissue interfaces, white matter, and hemorrhage degrades the ability to distinguish blood vessels in MR venography, as shown in Figure 4D. In contrast, this issue is not present in 3D gas ΔR_2_*-mMRA, as shown in Figure 4C.

### Comparison of 3D Gas ΔR_2_*-mMRA and 3D ΔR_2_-mMRA

**Figure 5:**
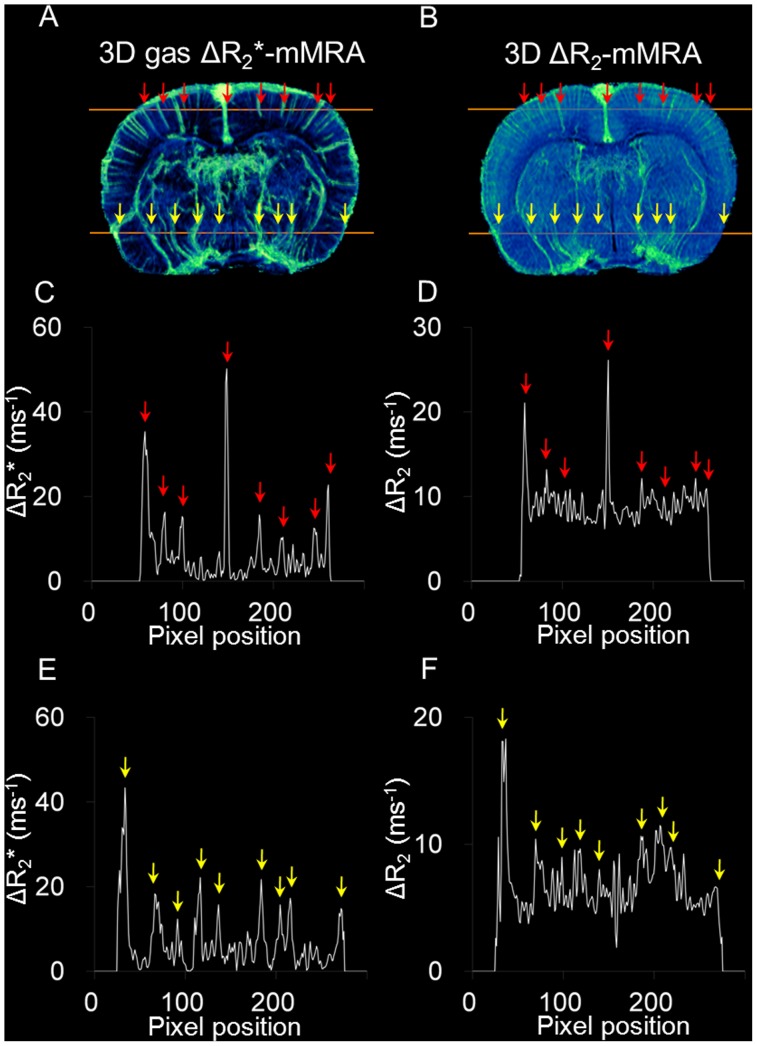
Comparison of 3D gas ΔR_2_*-mMRA and 3D ΔR_2_-mMRA. (A) An axial view from 3D gas ΔR_2_*-mMRA. (B) An axial view from 3D ΔR_2_-mMRA with identical geometrical settings in the same animal. The four sets of arrows label the vessels identified by both methods at the dorsal and ventral portions of the brain. (C, D, E, F) Line profiles, with the positions of vessels indicated by arrows.

The images obtained by 3D gas ΔR_2_*-mMRA and 3D ΔR_2_-mMRA using MION as the contrast agent were compared with identical resolution and geometrical settings in the same animal. Figure 5A and 5B show the results of 3D gas ΔR_2_*-mMRA and 3D ΔR_2_-mMRA for the same axial view, respectively. The microvasculature revealed by 3D gas ΔR_2_*-mMRA corresponds well with that revealed by 3D ΔR_2_-mMRA, although distinct features are evident. The arrows in the figures label the vessels identified by both methods at the dorsal and ventral portions of the brain. Line profiles of the vessels are shown in Figure 5C, 5D, 5E, and 5F. The profiles from 3D ΔR_2_-mMRA exhibited higher levels and more fluctuating peak heights relative to those from 3D gas ΔR_2_*-mMRA. The minimal baseline levels and consistently elevated peak heights in 3D gas ΔR_2_*-mMRA indicate a better vessel-to-tissue contrast.

**Figure 6:**
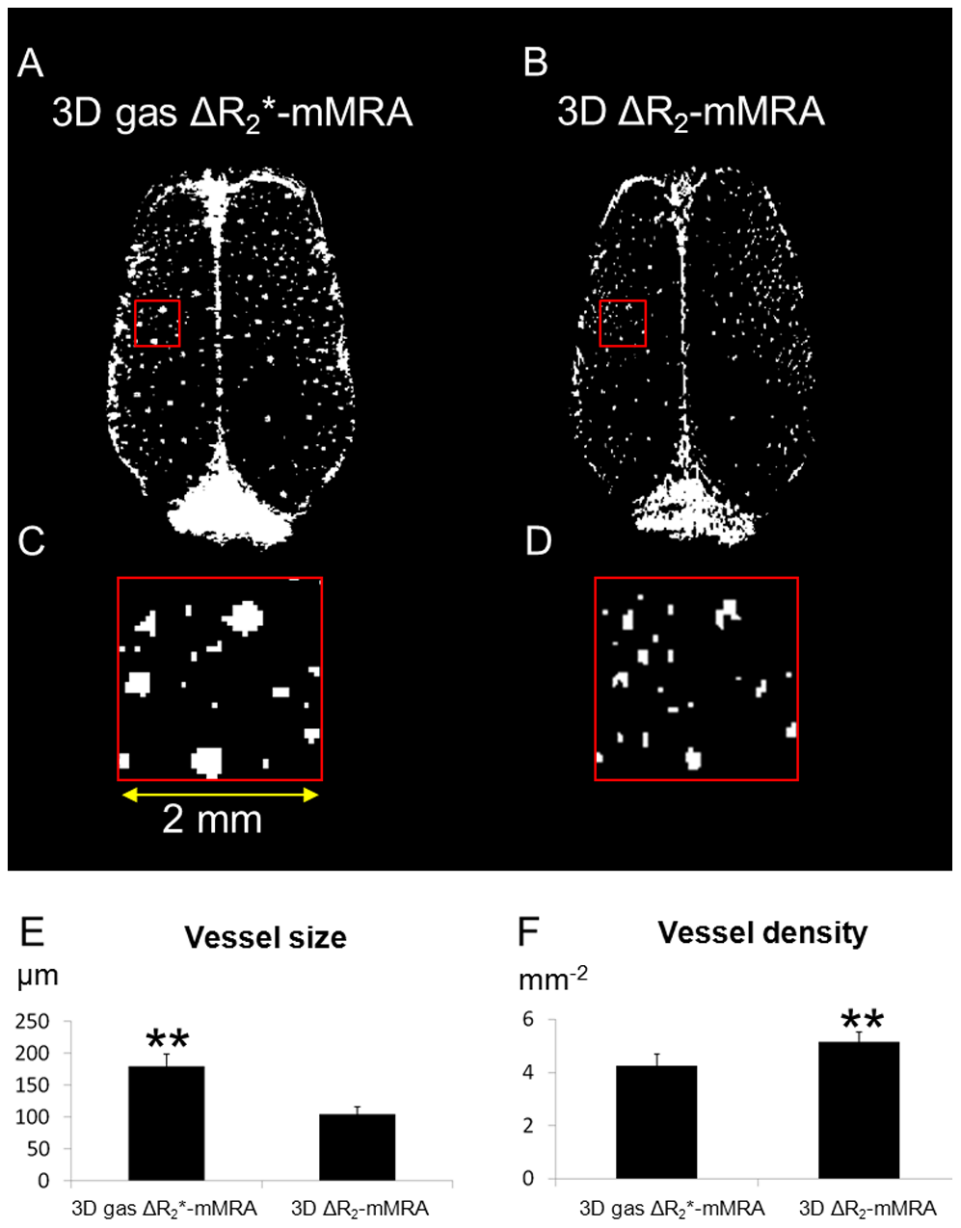
Differences in the characterized microvasculature between 3D gas ΔR_2_*-mMRA and 3D ΔR_2_-mMRA. (A) A 2×2 mm^2^ cortical slice in the horizontal plane from the ΔR_2_* map of 3D gas ΔR_2_*-mMRA. (B) The ΔR_2_ map of 3D ΔR_2_-mMRA. (C) Magnified view of the region of the ΔR_2_* map marked by the square in A showing bright signals representing the through-plane cortical penetrating vessels. (D) The bright signals were smaller but more numerous in the ΔR_2_ map. (E) Quantification of the sizes of vessels detected by the two methods. (F) Quantification of the densities of vessels detected by the two methods. Data in E and F are mean and SD values.

Cortical slices in the horizontal plane from a ΔR_2_* map of 3D gas ΔR_2_*-mMRA and a ΔR_2_ map of 3D ΔR_2_-mMRA are shown in Figure 6A and 6B, respectively. The distributions of the blood vessels revealed by the two methods are generally consistent, but fewer vessels were identified and the vessels appeared larger in 3D gas ΔR_2_*-mMRA relative to 3D ΔR_2_-mMRA. Figure 6C and 6D show enlarged views, while Figure 6E and 6F show quantifications of the size and density, respectively.

### Application of 3D Gas ΔR_2_*-mMRA to Study Poststroke Revascularization

**Figure 7:**
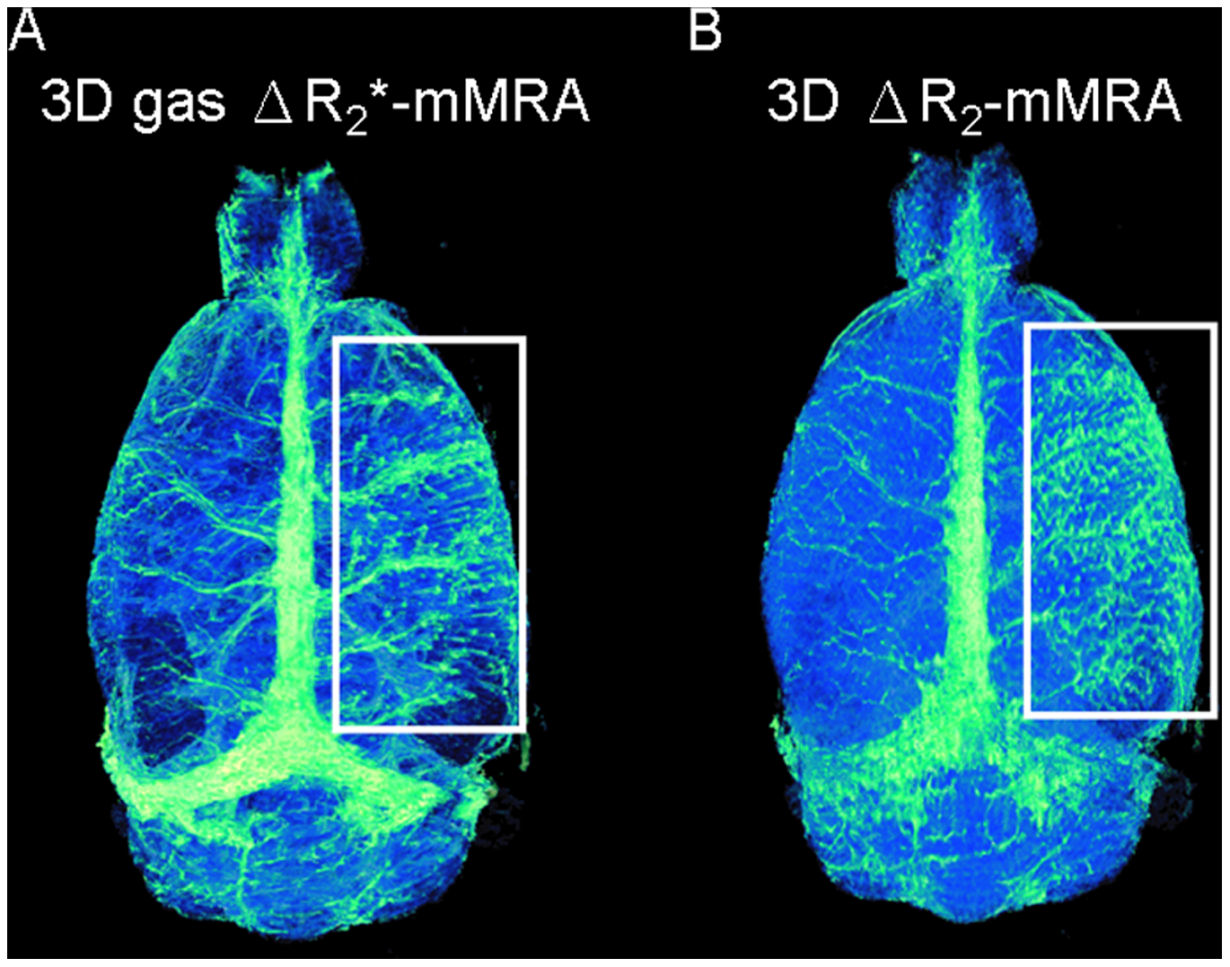
3D gas ΔR_2_*-mMRA applied to detect poststroke revascularization at 3 days after reperfusion. (A) 3D gas ΔR_2_*-mMRA shows an increased number of cortical vessels in the lesioned cortex (marked by the rectangle) relative to the unlesioned side. (B) 3D ΔR_2_-mMRA reveals a different microvasculatural pattern that is very likely confounded by the extravasation of the contrast agent due to the increased vascular permeability.

3D gas ΔR_2_*-mMRA and 3D ΔR_2_-mMRA were applied to a stroke rat at 3 days after reperfusion to study poststroke revascularization. Figure 7A shows that 3D gas ΔR_2_*-mMRA revealed an increased number of cortical vessels in the lesioned cortex (marked by a rectangle) relative to the unlesioned side. But this feature is difficult to see in 3D ΔR_2_-mMRA, as shown in Figure 7B, because the increased vascular permeability caused extravasation of the contrast agent, and thus resulted in inaccurate ΔR_2_ estimations in the region.

## Discussion

The present study demonstrates a new mMRA approach called 3D gas ΔR_2_*-mMRA that allows the visualization of cerebral microvessels with flexible viewing in various planes as well as providing quantitative information on rCBV. The high-resolution 3D imaging protocol and manipulation of the BOLD contrast by gas challenges are keys to this method. The vessels revealed by this method are predominantly veins and venules. This method provides a high vessel-to-tissue contrast that makes it easy to distinguish the microvasculature. The vessel sizes appeared magnified due to the susceptibility effect. When applied to studying poststroke revascularization, the method vividly reveals the microvascular remodeling changes at 3 days after reperfusion. This approach integrates the advantages of MR venography in using the intrinsic BOLD contrast and 3D ΔR_2_-mMRA in terms of high resolution and quantification.

Visualization of the cerebral microvasculature is a vigorous research area among many MR scientists and biologists. The acquisition of high-resolution signals is essential for achieving this goal. Previously developed methods can be categorized by the signal sources employed, including the flow effect, exogenous T_1_ or T_2_ contrast agents, and the intrinsic BOLD contrast. The flow effect provides the fundamental contrast giving rise to the visualization of major arteries by time-of-flight (TOF) MRA. However, it is difficult to use this effect to visualize microvessels due to the small flow effect [29]–[31], even when employing adjusted positioning and modified pulse sequences–the flow effect is not inherently useful as a signal source for visualizing the microvasculature.

Gadolinium-based T_1_ contrast agents can be used to optimize TOF-MRA [29]. This method of contrast-enhanced (CE) MRA enables the visualization of many more arterial branches than does TOF-MRA because of the presence of the contrast agent in the cerebral vasculature. However, CE-MRA-based imaging of microvessels is limited by the rapid washout of the contrast agent, which produces a trade-off between the short circulation time window of the contrast agent and the capability of high-resolution imaging [32].

Iron-based T_2_ contrast agents have been used in combinations of high-resolution T_2_ or T_2_* imaging protocols to visually detect even microvessels [5], [8], [33]. The long half-life of these T_2_ contrast agents in the circulation makes them an ideal signal source. Such an agent is injected intravenously to flow in the bloodstream–including in the arteries, arterioles, veins, venules, and capillaries–with a half-life of several hours, which enables the entire cerebral microvasculature to be visualized. The utility of these methods has been demonstrated in vascular remodeling, reorganization, and angiogenesis [5], [8], [33], [34]. Although the imaging results are satisfactory, certain practical hurdles restrict the further application of these methods: (1) the availability of iron-based contrast agents is becoming restricted, since they are being progressively removed from the market, (2) a high dose of the agents is needed to achieve the visualization, which may induce side effects in the subjects, and (3) iron-based contrast agents are expensive. These drawbacks severely hinder the potential use of 3D ΔR_2_-mMRA in clinical settings.

The use of the intrinsic BOLD contrast is an attractive alternative to either a T_1_ or T_2_ contrast agent, with the main advantage being that it is not affected by damage to the BBB [35]. A leaky BBB is a well-recognized phenomenon in brain disorders, including stroke, tumor, and Alzheimer’s disease. The extravasation of the contrast agent has at least three drawbacks: (1) suboptimal vessel-to-tissue contrast, (2) the signals caused by the leaked contrast agent being erroneously identified as vascular contrast, and (3) the consequent inaccurate, overestimated depiction of the cerebral microvasculature. The extravasation issue should be taken into account in any MRA or mMRA technique that uses exogenous contrast agents for signal sources, and CE-MRA and 3D ΔR_2_-mMRA inevitably inherit this disadvantage.

Gas-challenged BOLD contrast is a robust signal source for microvascular imaging [16], [17], [19]. This concept has been demonstrated previously in both the normal and pathological brain [19], [36], [37]. The choice of gases is critical to generating an adequate BOLD contrast difference. The sO_2_ levels under hypoxia, normoxia, hyperoxia, and hypercapnic hyperoxia vary from low to high [16], [38]. Cai et al. showed that the BOLD contrast between hypoxia and normoxia was sufficient for microvascular detection, but the use of gases with low oxygen levels can present risks to the subjects [19]. In contrast, hyperoxic gases such as 100% oxygen and carbogen are relatively safe and clinically applicable [16], and the present study chose the difference between normoxia and hypercapnic hyperoxia to produce the microvasculatural map. This paradigm of gas challenge should have only mild effects on the subjects, making it more suitable for clinical situations. Additional advantages of the gas BOLD contrast include its low cost, repeatability, and ease of timing. These features make 3D gas ΔR_2_*-mMRA very appealing for clinical use.

A practical concern when applying 3D gas ΔR_2_*-mMRA in clinical situations is the image acquisition time. Dynamic susceptibility contrast (DSC) MRI can assess the CBV [39], and vessel-size imaging can even provide an index of vessel size based on the ratio of ΔR_2_* and ΔR_2_ values much more rapidly [36], [37], [40], [41]. However, DSC-MRI and vessel-size imaging have the drawback of not providing information about the morphology of microvessels. Techniques such as parallel imaging, half-Fourier imaging, and segmented echo-planar imaging–which were designed to obtain better signal-to-noise ratio and resolution when using an acceptable acquisition time–would be helpful in adapting 3D gas ΔR_2_*-mMRA to clinical situations [42]–[44].

The BOLD 3D gas ΔR_2_* signals are dominated by the venous system, and 3D gas ΔR_2_*-mMRA provides three advantages over conventional MR venography. First, the boundary of vessels can be easily distinguished from the background in regions at air–tissue interfaces and near white matter based on higher ΔR_2_* signals, because the deoxyhemoglobin concentration in the background does not change during the gas challenge. However, the MR venography signals are obscured in those regions because of the similar hypointensities of vessels and background. Second, the gas-challenged BOLD contrast can provide better sensitivity in detecting hypoxic vessels in tumor or stroke conditions, since the accumulation of deoxyhemoglobin provides large ΔR_2_* signals [45], [46]. Third, 3D gas ΔR_2_*-mMRA can provide quantitative information about rCBV and reveal the microvessel morphology.

Ogawa and Lee reported that the susceptibility effect resulted in visualized vessels appearing twice their normal sizes in gradient-echo images compared to spin-echo images [47]. In the present study the observed vessel size in 3D gas ΔR_2_*-mMRA (∼200 microns) was also nearly twice that seen in 3D ΔR_2_-mMRA (∼100 microns) under our experimental conditions. This magnification is caused by the extravascular dephasing component that depends on TE, field strength, vessel orientation, and voxel size [47], [48]. Note that the resolution applied in this study was 50×50×73 microns, which is larger than the branches of intracortical vessels (<40 microns) [49], so the partial volume effect would cause the vessel size to be overestimated in both 3D gas ΔR_2_*-mMRA and 3D ΔR_2_-mMRA. Despite the susceptibility-effect-induced magnification of the observed vessel size, intracortical vessels with diameters as small as <80 microns reported by Park et al. can still be visualized by 3D gas ΔR_2_*-mMRA [15].

The BOLD contrast is sufficiently sensitive to allow the detection of even capillary signals [35]. The red blood cells that carry oxygenated or deoxygenated hemoglobin have diameters of approximately 8 microns in humans and 6 microns in rodents. The deformation abilities of the red blood cells enable them to pass through even the smallest capillary lumen of the microvasculature, which is ∼4 microns in humans and <3 microns in rodents (for reference, the size information relevant to this study is summarized in Table 2). Unfortunately, the direct visualization of capillaries by MRI remains unlikely irrespective of the angiographic method used due to its limited spatial resolution. To our knowledge the best resolution provided by high-field small-animal MRI is 78 microns in living rodents [15], and such resolution is insufficient for depicting the capillaries. Alternatives such as indirect capillary characterization are probably the only solution to acquire information from capillaries; for example, the rCBV measured using 3D gas ΔR_2_*-mMRA contains the capillary information.

**Table 2:** Size information of cerebral vascular components in humans and rodents in the literature.

Vasculature (µm)	Humans	Rodents
Macrovessels	>200 [48]	>100 [53]
Microvessels	<200 [48]	<100 [53]
Arterioles and venules	8–200 [50]	30–100 [53]
Capillaries	4–8 [51]	3 [54]
Red blood cell	7.82 [52]	6 [55]

## Conclusions

Cerebral microvascular abnormalities and the subsequent remodeling in brain disorders are important issues that remain less explored in studies of neurological disorders. The new method of 3D gas ΔR_2_*-mMRA presented here utilizes gas-challenged BOLD contrast to assess rCBV and directly visualize the morphology of cerebral microvasculature in rat brains, which offers the opportunity to thoroughly understand both the structural and functional characteristics of microvascular alterations in brain disorders. The advantageous features of minimal invasiveness, high vessel-to-tissue contrast, and not being affected by leakage problems make the method an appealing option for both fundamental research and clinical examinations.

## Supporting Information

Figure S1 3D gas ΔR_2_*-mMRA applied to investigate the poststroke revascularization. 3D gas ΔR_2_*-mMRA revealed few vessels in the ischemic lesion at 30 min and 1 day after reperfusion, while numerous vessels penetrating from the brain surface appeared at 3 days after reperfusion, as indicated by the red arrows.
(TIF)
