# Supplementary figures and images for: High-Resolution Structural and Functional Assessments of Cerebral Microvasculature Using 3D Gas ΔR2*-mMRA
Source: PLoS One. 2013 Nov 4;8(11):e78186. doi: 10.1371/journal.pone.0078186 (PMC3817180; doi:10.1371/journal.pone.0078186)

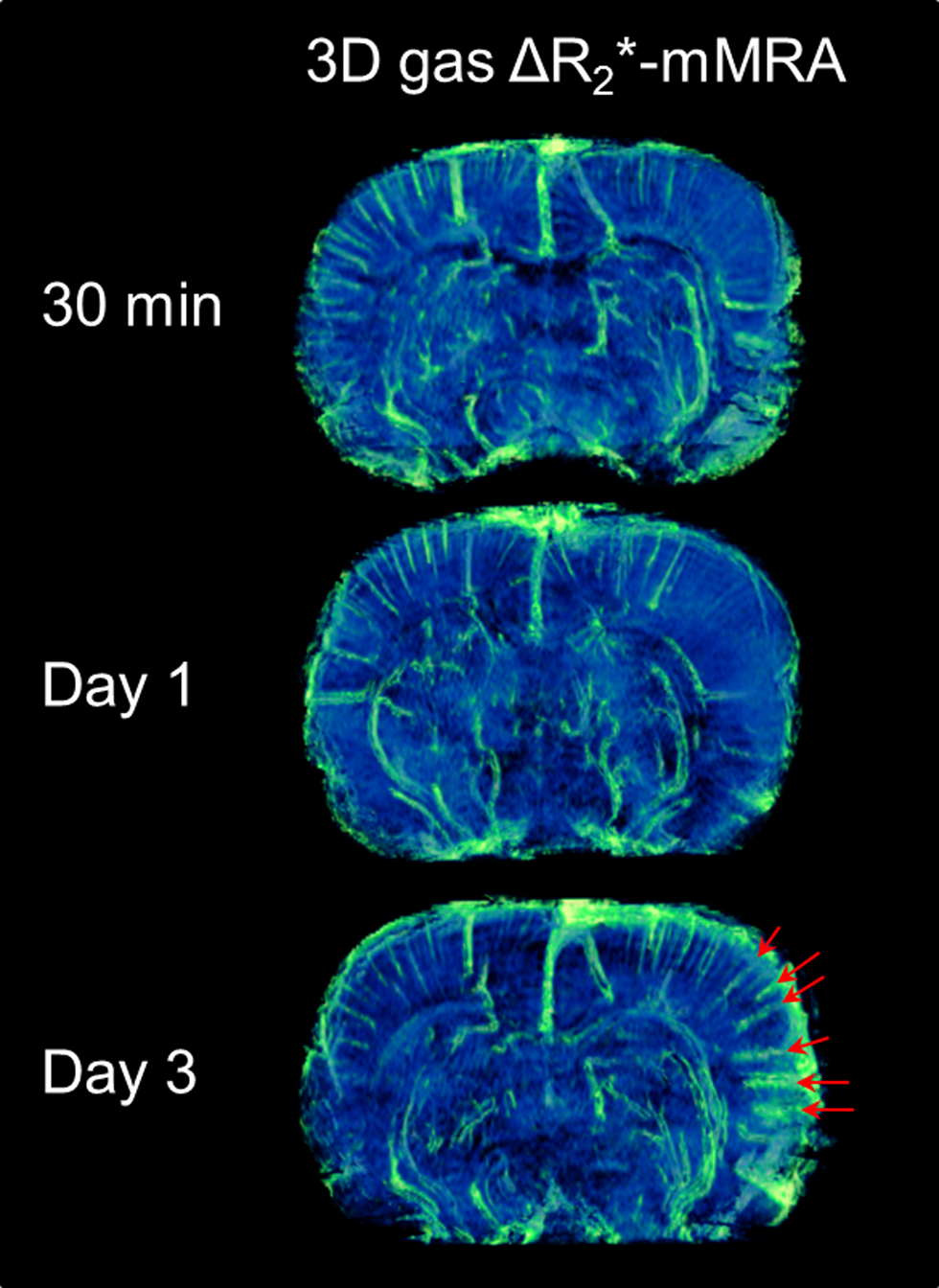

Supplement: Figure S1 — 3D gas ΔR2*-mMRA applied to investigate the poststroke revascularization. 3D gas ΔR2*-mMRA revealed few vessels in the ischemic lesion at 30 min and 1 day after reperfusion, while numerous vessels penetrating from the brain surface appeared at 3 days after reperfusion, as indicated by the red arrows. (TIF) [file pone.0078186.s001.tif]
